# Depression as a Risk Factor for Alzheimer’s Disease: A Systematic Review of Longitudinal Meta-Analyses

**DOI:** 10.3390/jcm10091809

**Published:** 2021-04-21

**Authors:** Olalla Sáiz-Vázquez, Patricia Gracia-García, Silvia Ubillos-Landa, Alicia Puente-Martínez, Silvia Casado-Yusta, Beatriz Olaya, Javier Santabárbara

**Affiliations:** 1 Department of Occupational Therapy, Faculty of Health Science, University of Burgos, C/Paseo de los Comendadores, Hospital Militar, 1, 09001 Burgos, Spain; osaiz@ubu.es; 2 Psychiatry Service, Hospital Universitario Miguel Servet, 50009 Zaragoza, Spain; pgraciag@salud.aragon.es; 3 Department of Social Psychology, Faculty of Health Science, University of Burgos, C/Villadiego, 1, 09001 Burgos, Spain; 4 Department of Social Psychology, Faculty of Health Science, University of Burgos, C/Paseo de los Comendadores, Hospital Militar, 1, 09001 Burgos, Spain; alicia.puente@ehu.es; 5 Department of Applied Economy, Faculty of Economics and Business Sciences, University of Burgos, Pza. De la Infanta Dª Elena, 09001 Burgos, Spain; scasado@ubu.es; 6 Research, Innovation and Teaching Unit, Parc Sanitari Sant Joan de Déu, Universitat de Barcelona, Carrer Doctor Pujadas 42, 08830 Sant Boi de Llobregat, Spain; beatriz.olaya@pssjd.org; 7 Centro de Investigación Biomédica en Red de Salud Mental (CIBERSAM), Ministry of Science and Innovation, Av. Monforte de Lemos 3-5, Pabellón 11, Planta 0, 28029 Madrid, Spain; jsantabarbara@unizar.es; 8 Department of Microbiology, Pediatrics, Radiology and Public Health, University of Zaragoza, C/Domingo Miral s/n, 50009 Zaragoza, Spain; 9 Aragonese Institute of Health Sciences (IIS Aragón), 50009 Zaragoza, Spain

**Keywords:** depression, Alzheimer’s disease, clinical and symptomatic criteria, meta-meta-analysis

## Abstract

Alzheimer’s disease (AD) is the most frequent cause of dementia, linked to morbidity and mortality among elderly patients. Recently, several clinical studies suggested that depression is a potential risk factor for cognitive decline and AD. A review of meta-analyses was performed, calculating pooled odds ratios to estimate the risk of AD in people with a prior diagnosis (or clinically significant symptoms) of depression. A total of six meta-analyses which represented 28 individual studies were analyzed. A significant association between depression and AD was found (OR = 1.54, 95% CI [1.02–2.31]; *p* = 0.038). The results showed that heterogeneity across studies was substantial. We found a significant positive effect size for clinical measures of depression, but not for symptomatic rating scales, in the association of depression with risk of AD. The type of rating scale used to assess depression and the cut-off criteria selected also moderated the relationship between depression and AD risk. We found that studies that used clinically significant criteria for diagnosis of depression had more consistent and significant results than studies that used symptomatic scales.

## 1. Introduction

Alzheimer’s disease (AD) is the most frequent cause of dementia and is considered one of the main causes of morbidity and mortality among elderly people [1]. The World Alzheimer’s Report revealed that 46.8 million people worldwide were living with dementia in 2015, and the total global social cost of dementia was estimated to be $818 billion [2]. Estimates of dementia incidence in population-based studies range from 5 to 10 cases per 1000 person-years in people aged 64 to 69, and up to 40 to 60 cases per 1000 person-years in people aged 80 to 84 [3]. In 2017 in Europe, prevalence rates of AD were reported to be 5.05%, with 3.31% in men and 7.13% in women [4]. Given the personal and social consequences of dementia and AD demand, we accelerate the global effort to understand this complex disorder [5].

Decades of research revealed that the pathophysiological mechanisms underlying this neurodegenerative disease include accumulation of amyloid-beta peptide (Aβ) in brain tissues and cytoskeletal changes related to the hyperphosphorylation of microtubule-associated Tau protein in neurons. As a consequence, neuritic plaques and neurofibrillary tangles are accumulated, mostly in the medial temporal lobe and associative neocortical areas [6], and resulting in several cognitive deficits. The clinical manifestation of AD is progressive, from unnoticeable brain changes to brain changes that cause cognitive deterioration and eventually physical disability [7]. AD usually begins with memory difficulties followed by other cognitive problems such as visuospatial abnormalities, navigation difficulties, executive problems, and language disturbance [2].

Evidence seems to suggest that the etiology of AD is multifactorial, with genetics, older age, and a family history of AD being the greatest contributors to a higher risk of AD [7]. Furthermore, AD is often associated with other chronic diseases (diabetes, cholesterol, cardiovascular diseases, obesity, and hypertension) [8]. Although these risk factors are unchangeable, other risk factors can be modified to reduce the risk of dementia and cognitive decline. This is particularly important, since there is no currently available way to stop the damage and destruction of neurons linked to AD.

Depressive symptoms are common in AD and occur in approximately 20–30% of patients [9]. Depression is a serious medical illness that affects about 300 million people worldwide and which might aggravate existing medical conditions and increase functional disability [9,10]. Clinical evidence suggests a relationship between depression and AD [11,12,13,14]. However, it remains unclear whether depression represents a risk factor for AD, is an early symptom of neurodegeneration, or is a reaction to early cognitive deficits [14,15]. Some studies have suggested that depressive symptoms immediately follow the onset of AD rather than precede it [16]. Moreover, evidence from other studies indicates that depression has only a mild effect on dementia [17] and does not increase the risk for developing AD [18]. However, other authors suggest that the presence of depression in patients with AD increases the risk of behavioral disturbance and accelerates functional decline [12]. Hudon et al. [19], for example, found that depression was the most consistent risk factor associated with behavioral or psychological symptoms and cognitive decline in patients with AD. In addition, several studies concluded that late-life depression is related to an increased risk for all-cause dementia, vascular dementia, and Alzheimer’s disease [20,21,22], and late-life depression was shown to be associated consistently with a two-fold increased risk of dementia [23,24].

In order to clarify the role of depression as a risk factor of AD, several meta-analyses were conducted [19,20,22,23]. However, some limitations were pointed out. Cherbuin et al. [24], for example, indicated that, in general, results from previous studies that focused on depression as a risk factor of AD might be biased due to the type of instrument used to assess depression. Results are frequently based on different tools. Some of these studies are based on symptomatic rating scales with cut-off points (e.g., CESD), while others are based on clinical criteria (e.g., DSM). Thus, the pooled estimates of the risk for AD in depressed people might be unreliable, because these meta-analyses combined effect sizes from studies using different instruments to assess depression (i.e., symptomatic rating scales and clinical diagnoses). Additionally, these previous meta-analyses did not pool findings separately for studies using clinical criteria and studies using depressive symptom rating scales with specified cut-off points.

Based on these limitations and the inconclusive evidence, we aimed to perform a meta-meta-analysis of longitudinal studies to assess the effect of depression on the risk of a subsequent diagnosis of AD. Given the expected heterogeneity among studies, we also aimed to pool findings separately from studies using clinical criteria and those using depression symptom rating scales, and to test the association between depression and risk of AD according to the different instruments used.

## 2. Materials and Methods

### 2.1. Data Collection

This meta-meta-analysis was performed in accordance with the Preferred Reporting for Systematic Reviews and Meta-analysis (PRISMA) Statement [25]. For data collection, we searched meta-analyses that measured depression at baseline and reported outcomes in individuals with diagnoses of AD at follow-up. ISI Web of Science (WOS), Scopus, Pubmed, Elsevier Science Direct, and Google scholar were searched from inception up to 31 July 2020. Combinations of the following search terms were used: “depression” AND “Alzheimer’s disease” AND “meta-analysis”. The data search was done in English (four studies) and Spanish (one study). When necessary, corresponding authors were contacted to provide full text details of the study outcome measures.

### 2.2. Inclusion Criteria

By consensus of the authors, studies were included if they met the following criteria:

1. Longitudinal studies that investigated the effect of depression or depressive symptoms (at baseline) as an antecedent to AD (follow-up).
2. Studies including patients with a diagnosis of AD according to diagnosis criteria (e.g., Related Disorders Association criteria, N-ADRDA, the Diagnostic and Statistical Manual of mental Disorders, DSM-III or the National Institute of Neurological and Communication Disorders-Alzheimer’s Disease).
3. Studies that clinically assessed levels of depression by means of a clinical diagnosis (e.g., DSM-IV, ICD-10), or a symptomatic diagnostic tool with a cut-off score (e.g., Geriatric Mental State Schedule, GMS) that identifies clinically significant levels of depression.
4. Studies reporting sufficient information to calculate common effect size statistics (i.e., mean and SD, exact P-, t-, or z-values).
5. Original, peer-reviewed meta-analyses that were published in English and Spanish.

### 2.3. Exclusion Criteria

By consensus of the authors, the following were excluded:

1. Studies investigating the association of depression and risk of AD using a sample of patients with AD and other dementia (non-independent or overlapping data for AD).
2. Studies not reporting quantitative data to calculate the association between depression and AD, or not published as meta-analyses in peer-reviewed journals (i.e., conference abstracts, book chapters).
3. Meta-analyses about other topics or those that included the same primary studies.

### 2.4. Data Extraction and Quality Assessment

Titles and abstracts of potential meta-analyses about depression and incident AD were independently analyzed by three researchers (OS, SU, PG). After exclusion of irrelevant articles, the remaining meta-analyses were critically inspected to check data accuracy. Then, full texts of all primary studies included in each meta-analysis were screened according to the inclusion criteria. In the event of ambiguity, two authors (SU, JS) additionally reviewed the study to reach a consensus regarding its eligibility.

Data related to the diagnosis/assessment of depression and AD were collected directly from the text or from statistical tables. The lead author and either the third or fourth author independently extracted data from each study, including study characteristics (year, country, total sample size, and length of follow-up period), sample characteristics (mean age, % of women), measures of depression and AD, and the cut-off point used for depression in each individual study.

Diagnoses of AD were based on the following accepted clinical criteria: Revised criteria and the National Institute of Neurological and Communication Disorders-Alzheimer’s Disease and Related Disorders Association criteria (N-ADRDA), the Diagnostic and Statistical Manual of Mental Disorders in different editions (DSM-III, DSM-III-R, DSM-IV, DSM-V), and the International Classification of diseases (ICD-10). Additionally, studies established different cut-off scores on neuropsychological tests for the purposes of screening out cognitive impairment and dementia at baseline (see Table 1). Participants with scores above the cut-off on cognitive domains were excluded on the basis that this level of test performance indicates the presence of dementia or cognitive impairment. The most frequently used measures to describe the cognitive characterization of the participants at baseline were the Mini Mental State Examination (MMSE) (*n* = 14) and the Clinical Rating Scale (CRS) (*n* = 6). Diagnoses of depression were based on either symptomatic rating scales or clinical diagnoses. Clinical criteria for depression included the DSM-III, DSM-III-R, DSM-IV, DSM-V, and the Automated Geriatric Examination for Computer Assisted Taxonomy (GMS-AGECAT). Diagnoses of depression were based on symptomatic rating scales on valid cut-off points (SGDS/15/30, CES-D/10/11/20, HRSD-17).

In addition, the quality of the included studies was reported using the Assessment of Multiple Systematic Reviews (AMSTAR) tool [26], which was previously shown to have good inter-rater agreement, reliability, and content validity [26,27].

### 2.5. Statistical Analysis

Crude odds ratios (ORs) (and 95% confidence intervals (CIs)) were used to calculate the risk of developing AD associated with previous depression. When the number of cases of depression and AD were not provided, the effect sizes were calculated using reported data in the meta-analysis according to Lipsey and Wilson [28]. We considered HRs and ORs as equivalent, since it was previously shown that for rare events, they can be considered equivalent (incidence < 15%) [29]. Seventeen studies provided data that could be used in calculating crude ORs (odds of an outcome in the intervention arm divided by the odds of an outcome in the control). Eleven additional studies provided data on AD risk in samples as HR or ORs with 95% confidence intervals that could be used in pooling estimates.

Summary statistics were calculated using Comprehensive Meta-Analysis software (CMA; Version 3) (Biostat Inc., Englewood, NJ, USA) [30,31]. Initially, we performed an analysis summarizing all data available, including all studies with validated cut-offs or clinical diagnoses in a single pooled estimate [31]. For each study, we calculated: (a) 95% CI of the effect, (b) Z value and p (two-tailed significance), and (c) k or number of studies [32]. Presence of publication bias was assessed through visual inspection of funnel plots and with Egger’s test [16].

The level of heterogeneity was assessed with the I2 statistic, which describes the percentage of total variation across studies due to heterogeneity rather than chance alone. An I2 value of 25% indicates low heterogeneity, 50% moderate heterogeneity, and 75% high heterogeneity [31]. Random-effect models were used to determine statistically significant heterogeneity. Additionally, the Cochran Q test was applied to assess significant heterogeneity (*p*-value < 0.05). Moderating variables were selected on the basis of substantive considerations and the availability of data across studies included in the meta-analysis. Subgroup analyses were performed according to how depression was assessed: by clinical diagnosis (e.g., DSM-V) or by symptomatic rating scales (e.g., CES-D). Additionally, because the studies included different symptomatic rating scales, we also considered the instrument and the specific cut-off criteria as moderating variables. Therefore, we calculated the effect sizes of the association between depression and risk of AD separately for studies using different cut-off points. Finally, meta-regression analyses were conducted to obtain the proportion of variance explained for each moderator (the R-square analog). The scatter plot represents the mean effect for each level of covariate.

## 3. Results

**Figure 1:**
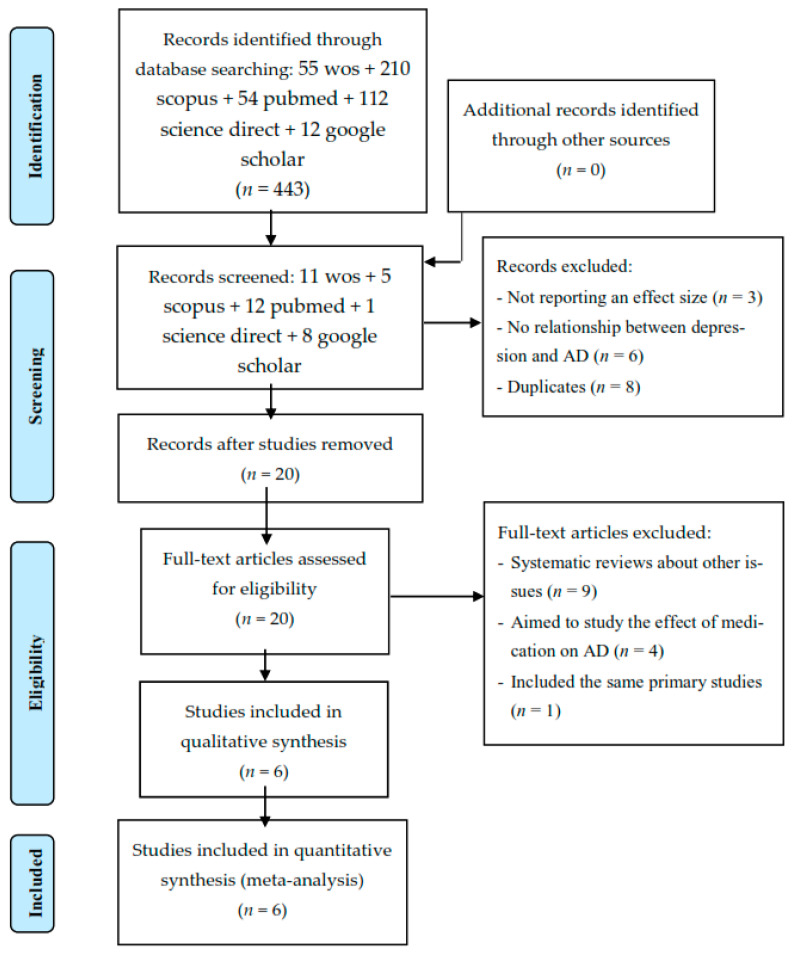
Flow chart depicting the selection of articles for our meta-analysis. Note: AD: alzheimer’s disease; n: number of studies.

The search strategy produced a total of 443 meta-analyses (see Table 1). Initially, 37 meta-analyses were eligible for inclusion. Of these, 31 were excluded: (a) 3 did not report an effect size; (b) 6 did not provide information on the relationship between depression and AD; (c) 8 were duplicates; (d) 9 were systematic reviews about other topics; (e) 4 aimed to study the effect of medication on AD; and (f) 1 included the same primary studies as another. Finally, a total of six meta-analyses were analyzed (k = 28 pooled effect sizes), representing data from *n* = 28 individual studies (see Figure 1).

Since the effect estimated from a biased collection of studies might overestimate the true effect, we assessed the likely extent of this bias and its potential impact on the conclusions. The result of Egger’s test was not significant: The intercept (B0) was 0.53, 95% CI (−1.88 to 2.95), with t = 0.45, df = 26, *p* = 0.65, indicating no publication bias.

### 3.1. Overall Results from the Meta-Analysis

A total of 28 individual studies reported the association between depression at baseline and AD at follow-up with a total of 101,881 participants (Nbaseline = 51,830; Nfollow-up = 50,051). Individual sample sizes ranged from 60 to 12,083. The majority of subjects was female. The mean age was 71.95, ranging from 52.7 to 81 years. One study did not report gender and age [53]. The mean follow-up length was 4.90 years (range from 1 to 23.6), with one study not reporting the number of years [54]. Characteristics of the 28 individual studies are presented in Table 1.

A total of 17 and 11 studies were based on symptomatic rating scales and clinical criteria to assess depression, respectively: CES-D (*n* = 14) (50%), DSM-III/III-R/IV/V (*n* = 8) (28.6%), GMS-AGECAT (*n* = 3) (10.7%), GDS (*n* = 2) (7.1%), and HAM-D (*n* = 1) (3.6%). AD diagnosis was established based on the N-ADRDA (*n* = 17) (56.7%) or DSMIII-R/IV/V (*n* = 10) (33.3%), ICD10 (*n* = 2) (6.7%), and N-AIREN (*n* = 1) (3.3%) scales.

**Figure 2:**
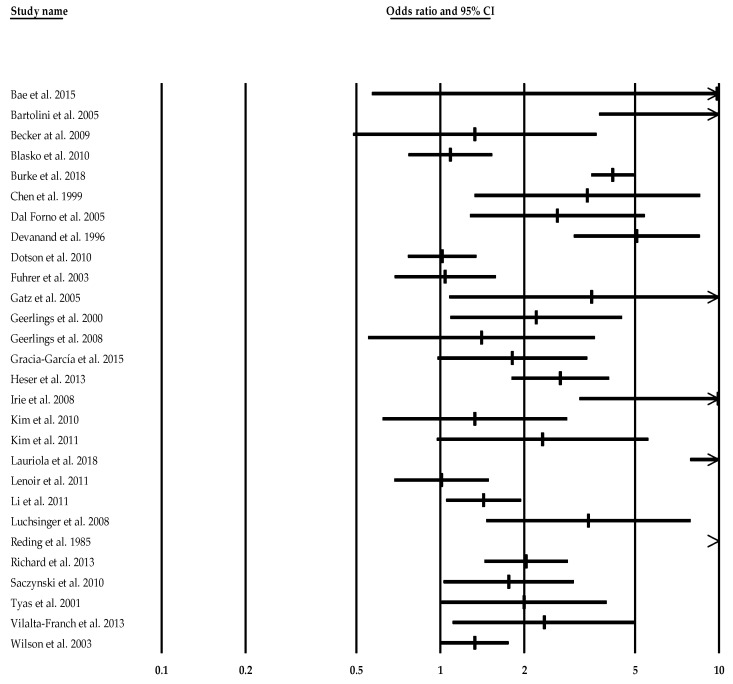
Forest plot of studies investigating the risk of Alzheimer’s disease (Time2) associated with depression (including all instruments).

Risk estimates were pooled across the 28 studies. The random effect of the relationship between depression and AD was significant (OR = 2.46, 95% CI [1.81–3.35], Z = 5.72, *p* < 0.001). Figure 2 shows the forest plot of the effect sizes and their 95% CI. Heterogeneity across studies was substantial (Q-value = 284.53, df = 27, I2 = 90.51, *p* < 0.001), suggesting the presence of potential moderators (Table 2).

### 3.2. Clinical Criteria and Symptomatic Rating Scales to Assess Depression

We tested three different models that reflected a combination of methodological moderators (see Table 3). Random effect models revealed a significant positive effect size of the association between depression and risk of AD for clinical (k = 11) and symptomatic (k = 17) measures of depression. Heterogeneity was substantial for the depression criteria (I2 = 90.51), indicating that the OR was greater for clinical than symptomatic measures.

Then, we performed an additional sub-group analysis distinguishing between types of symptomatic rating scale used to assess depression. The total effect (OR) was significant (1.80, 95% CI: 1.16–2.78, Z = 2.62, *p* = 0.009), and heterogeneity was moderate (I2 = 61.84). Sub-group analysis yielded a significant effect of depression on the development of AD for studies using the CES-D scales and HSRD, although this effect was non-significant when studies used the GDS scale. Only one study included the HSRD scale, and no additional subsample analyses were conducted. However, sufficient data were available for the CES-D (k = 14). We conducted further sub-analyses according to different cut-off points of the CES-D scale to define presence of depression. ORs were pooled across 14 studies (OR = 1.68, IC95% 1.24–2.27, Z = 3.36, *p* = 0.001). Heterogeneity was moderate across these studies (I2 = 63.95), indicating that the effect of depression on the risk of AD may differ according to the cut-off points used. Estimates were significant for ≥10 and ≥16 cut-offs, whereas the effect of depression on AD was not significant when studies used a cut-off of ≥4 and ≥20 (Table 3).

### 3.3. Meta Regression Analysis

We conducted a meta-regression analysis to determine whether the criteria used to measure depression might explain differences across studies in reporting effect size and might also cause heterogeneity. A significant negative effect of the use of symptomatic rating scales on the prediction of AD was found (b = −0.71, Se = 0.27, 95% CI: −1.24/−0.17, Z = −2.59, *p* = 0.009) compared to clinical criteria (k = 28, intercept: b = 1.30, se = 0.21, CI: 0.89/1.72, Z = 6.14, *p* ≤ 0.001) (Q = 6.71, df = 1, *p* = 0.009). Together, these explained 26% of the variance. That is, the use of symptomatic rating scales to assess depression was associated with a decreased likelihood of developing AD in the follow-up compared to the use of clinical criteria.

No significant moderating effects were found in meta-regression analyses conducted for the various symptomatic rating scales of depression (k = 17) (1 = GDS, intercept: b = 0.47, Se = 0.45 (−0.41/1.36), Z = 1.04, *p*= 0.296; 2. CES-D: b = −0.02, Se = 0.46 (−0.93/0.89), Z = −0.04, *p* = 0.97; 3. HSRD: b = 0.75, Se = 0.68 (−0.59/2.09), Z = 1.10, *p* = 0.270) (Q = 2.18, df = 2, *p* = 0.336). Differences explained the 28% of variation observed in the association between depression and AD.

**Figure 3:**
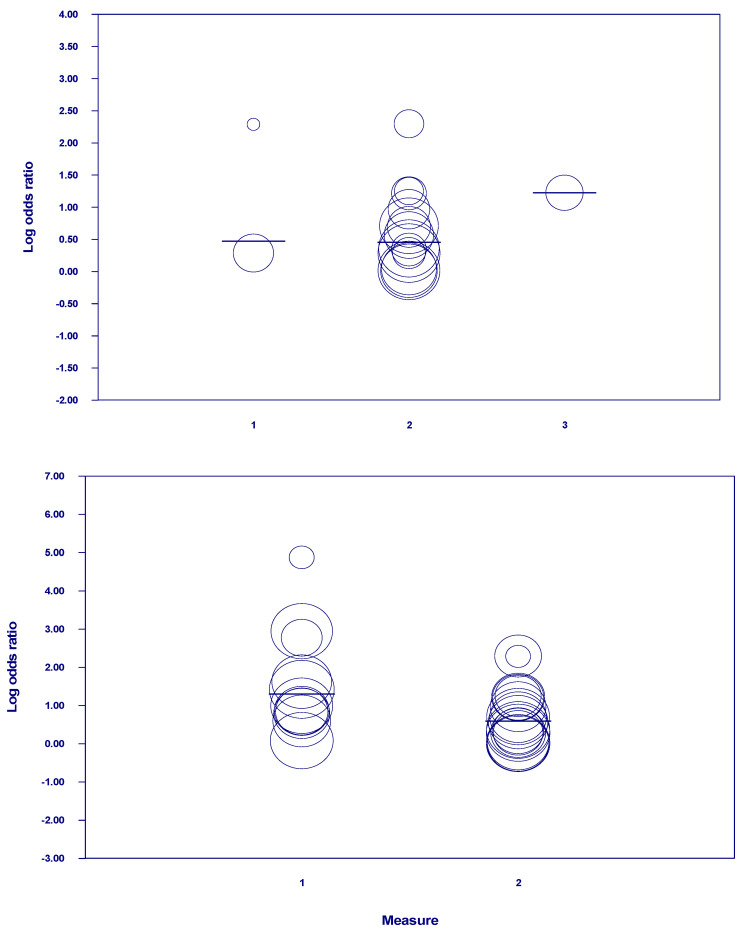
Meta-regression of log odds ratio on type of measure, symptomatic tool, and CES-D cut-offs (95% intervals are simultaneous and based on Z distribution). Scatterplots show the relationship between each covariate and AD.

When analyzing the differential effect of the CES-D cut-offs on the development of AD (k = 14), results showed a greater predictive effect for studies using more restrictive cut-off points (≥20) (intercept: b = 0.97, SE = 0.37, 95% CI: 0.25/1.69, Z = 2.63, *p* = 0.008) (≥4: b = −0.51, Se = 0.38 (−1.26/0.24), Z = 1.34, *p* = 0.180; ≥0.10: b = −0.50, SE = 0.39 (−1.27/0.28), Z = −1.26, *p* = 0.209; ≥0.16: b = −0.77, SE = 0.38 (−1.51/−0.01), Z = −2.04, *p* = 0.041) (Q = 7.43, df = 3, *p* = 0.050]. The different cut-off points of the CES-D explained the 53% of variation in the diagnosis of AD (Figure 3).

## 4. Discussion

The main contribution of this study was to produce precise AD risk estimates associated with different depression criteria, either clinically significant or based on symptomatic scales. Based on the results of 11 cohorts, we found a more than three-fold increased risk of AD for clinically significant depression. Likewise, based on findings of 17 cohort studies, the risk of AD increased almost two-fold in participants diagnosed with symptomatic measures of depression. We found that studies that used clinically significant criteria for diagnosis of depression had more consistent and significant results than those that used symptomatic scales.

However, most included studies used self-reported symptomatic scales for diagnosis of depression, specifically the CES-D. We further analyzed the differential effect of CES-D cut-off points on AD risk and found that they explained 53% of the variability of results. We found a slightly significant predictive effect in meta-analyzed data of studies using the cut-off CES-D point ≥10 and ≥16, but predictive risk of AD was greater for one study using a more restrictive cut-off point (≥20). Our results are consistent with those of Cherbuin et al. [24] who found that the meta-analysis of studies using a cut-off previously validated against clinical criteria (≥20) demonstrated higher risk estimates than those using a more lenient cut-off (≥16).

We found a greater effect of clinically significant depression on AD risk than the MA of Santabárbara et al. [21], probably because that meta-study included only three studies with homogeneous criteria for the diagnosis of depression (GMS-AGECAT). We also included eight studies using DSM criteria for depression; all of them but one, Blasko et al. [35], found consistently higher risk of AD compared to any other criteria. However, some of them found relatively large [34,53] or even extreme values of OR [49].

Furthermore, our study includes recent references [20], and it did not analyze data from studies of patient groups with mixed psychiatric histories or all types of dementia. Even though the meta-analysis of Kuring et al. [20] analyzed 36 independent studies for all types of dementia, they only pooled k = 8 studies for AD (OR = 2.23). This inclusion criterion may explain why our results show a greater OR risk from depression to AD than previous meta-analyses [20,21,22,23,24]. Furthermore, they did not analyze variability arising from the type of measure (clinical or symptomatic criteria) and from cut-off points used to assess depression across studies. Another strength of our study is that it includes a selection of prospective cohort studies to provide more evidence in establishing the cause and effect, and the relationship between depression and AD [21]. We analyzed a long follow-up period (4.9 years, range 1–23.6 years) to observe the potential association between depression (as an antecedent) and risk of AD, avoiding cross-sectional studies [20,24]. This analysis covers a gap in the previous literature, adding new information about the association between depression and AD. Finally, previous meta-analyses limited the literature search to biomedical databases. In our study, we included five databases in order to provide coverage of publications from different countries, reducing the likelihood of publication bias [22,23].

Overall, our study is the first to review all previously available meta-analyses of depression as a risk factor of incident AD systematically. Moreover, we included individual studies when they assessed clinically significant depression or a validated cut-off score in a symptomatic depression scale, and we conducted differential meta-analysis of specific AD risk estimates according to depression criteria. Our study demonstrates how depression criteria can explain variability between studies in the association between depression and incident AD. We agree with Cherbuin et al. [24] about the importance of using objective and specific measures of risk in evidence-based clinical practice.

A number of different hypotheses on the association between depression and dementia were suggested, yet the ways in which depression influences AD are as yet unclear. For instance, antidepressant use (i.e., anticholinergic drugs) was shown to be associated with an increased risk of dementia [68,69,70,71]. Furthermore, the ε4 allele of apolipoprotein E (APOE) was associated with the development of AD [46,47]. However, the idea that ε4 and dementia may be linked has little support [72,73,74,75]. In this vein, some risk factors, such as brain-vascular [76], cortisol, hippocampal atrophy [77], and neuroinflammation, could involve a possible common pathway to explain the association between depression and AD [78].

We should also recognize some limitations of our study. Firstly, as the studies included in the meta-analyses reported either the odds ratio or the hazard ratio for the association between late-life depression and dementia, we calculated the pooled OR for the association between depression and AD separately. Odds ratio is a measure of association between two conditions (such as in logistic regression models), whereas the hazard ratio is a measure of the strength of the association between two conditions in time-to-event statistical analysis. Given this, we should interpret the results from the pooled risk analysis with caution, as we included studies that reported hazard ratios and odds ratios together. Nonetheless, the results are very consistent across all analyses for AD. Secondly, individual studies assessing depressive symptoms by self-rating scales used pre-established cut-off scores, and no structured interviews were conducted for the diagnosis of depressive disorders, which may have introduced significant heterogeneity into the classification of cases and non-cases, in particular in individuals with mild depression; according our results, this may explain a good deal of the variability in results between studies. In addition, some of the studies included in this meta-analysis were not representative of the entire population (such as studies including only men) [46]. Although we did not find a moderator effect of observation time (results not shown), and the results support the hypothesis that clinical depression is a risk factor for later development of Alzheimer’s disease, the influence of prodromal symptoms should not be discounted, and it remains to be determined. Furthermore, we did not examine the influence of any single study on the overall risk estimates with sensitive analysis that omitted them one by one. Moreover, by choosing to include studies that allowed us to calculate crude ORs, we implicitly included studies that provided estimates of the relation between depression and AD risk in the form of unadjusted ORs, so other study-related factors may have affected the outcomes of these studies (age or sex). Inclusion of these studies may have biased our results. Another possible limitation of this meta-analysis is that our search was limited to certain databases. We did a careful review of all references in potentially relevant publications, previous meta-analyses, and systematic reviews published on depression and AD. Nevertheless, a search of other international databases (such as EMBASE and PsycINFO) might have led to the identification of additional studies that could have been included in this meta-analysis.

## 5. Conclusions

Although we cannot yet assert an etiological basis of the association, our study provides consistent data pointing to an increased risk of AD for clinically significant depression. Our findings highlight the importance of using more stringent and objective measures of depression in future studies. Depression should be assessed by clinicians with standardized, validated measures, and preventive strategies targeting at-risk individuals should be designed. Further studies need to assess the potential for treatment of clinically significant depression to decrease the risk of AD.

## Figures and Tables

**Table 1:** Summary of demographic and study information.

Study	Year	Country	AD Measure ^1^	Cognitive Measure ^2^	Cut-Off Criteria Cognition	Depression Measure ^3^	Cut-Off Criteria Depression ^4^	*n* ^5^	Follow-Up Length (Years) *M* (*SD*)	Age *M* (*SD*)	Female (*%*) (Total)	AMSTAR2 ^6^
Bae et al. [33]	2015	AS	N-ADRDA	CERAD-K	≥60	GDS15	≥8	540	3.5 (0.3)	71.7 (5.1)	55.2	HIGH
Bartolini et al. [34]	2005	EU	N-ADRDA	MMSE	>26	DSM-III-R	-	222	1	69.2 (4.8)	63.5	HIGH
Becker et al. [18]	2009	USA	N-ADRDA	MMSE	>26	CES-D20	≥10	729	7.1 (NR)	70	69	HIGH
Blasko et al. [35]	2010	Austria	N-ADRDA	CERAD	≥60	DSM-IV	-	648	2.5 (NR)	78.3 (0.5)	56.5	HIGH
Burke et al. [36]	2018	USA	N-ADRDA	CRS	≤3	DSM-V	-	12,083	4.2 (-)	63.9	83	HIGH
Chen et al. [37]	1999	USA	DSM-III-R	MMSE	>26	CES-D20	≥16	803	4.5 (NR)	73.7 (5.0)	60	MODERATE
Dal Forno et al. [38]	2005	USA	N-ADRDA	BIMC		CES-D20	≥20	1357	6.1 (-)	65.5 (12.0)	45.5	HIGH
Devanand et al. [39]	1996	USA	N-ADRDA	CRS	≤3	DSM-III R	-	456	2.54	72	70	HIGH
Dotson, Beydoun & Zonderman [40]	2010	USA	DSM-III R	BIMC		CES-D20	≥16	2177	23.6 (NR)	52.7 (18.8)	42.3	HIGH
Fuhrer, Dufouil & Dartigues [41]	2003	France	N-ADRDA/DSM-III-R	MMSE	>26	CES-D20	≥16	1576	8.0 (NR)	75.2 (6.9)	58.3	HIGH
Gatz et al. [16]	2005	Canada	DSM-III R	MMSE	>26	CES-D20	≥16	766	5	74.5 (6.0)	61.7	HIGH
Geerlings et al. [42]	2000	Países Bajos	DSM-III-R	MMSE	>26	GMS- AGECAT	-	1911	5,9 (1,6)	73.5 (7.9)	49	MODERATE
Geerlings et al. [43]	2008	Netherlands	N-ADRDA	MMSE	>26	CES-D20	≥16	393	5.9 (1.6)	73.5 (7.9)	49	MODERATE
Gracia-García et al. [44]	2015	EU	DSM-IV	MMSE	>26	GMS- AGECAT	≥3	3626	4.5	71.9 (9.0)	54.4	HIGH
Heser et al. [45]	2013	Germany	DSM-IV/ICD-10	MMSE	>26	DSM-IV	-	2969	4	81	64.8	HIGH
Irie et al. [46]	2008	USA	N-ADRDA	CRS	≤3	CES-D11	≥9	1585	5.1	76.3 (3.6)	0	HIGH
Kim et al. [47]	2010	South Korea	N-ADRDA	CRS	≤3	GDS30	13/14	473	2.4 (0.3)	71.8 (5.1)	54.4	HIGH
Kim et al. [48]	2011	South Korea	DSM-IV	CRS	≤3	GMS- AGECAT	≥3	563	2.4 (0.3)	71.8 (5.0)	54.4	MODERATE
Lauriola et al. [49]	2018	EU	DSM-V	MMSE	>26	DSM-V	-	181	4	74.5 (7.5)	59.7	HIGH
Lenoir et al. [50]	2011	France	N-ADRDA	MMSE	>26	CES-D20	M ≥ 16 W ≥ 22	7989	4 (NR)	74.0 (5.4)	61.3	HIGH
Li et al. [51]	2011	USA	N-ADRDA	CASI	≥78	CES-D11	≥10/	3410	7.1 (NR)	74.9 (6.2)	59.9	HIGH
Luchsinger et al. [52]	2008	USA	N-ADRDA	CRS	≤3	HRSD17	≥10	1138	5.1 (3.3)	75.1 (6.4)	67.7	HIGH
Reding, Haycox & Blass [53]	1985	USA	ICD-10	MSQ	0–2 errors	DSM-III	-	60	3	-	-	MODERATE
Richard et al. [54]	2013	USA	DSM-III R	MMSE	>26	CES-D10	≥4	2160	-	76.9 (7.1)	75	MODERATE
Saczynski et al. [55]	2010	USA	N-ADRDA	MMSE	>26	CES-D20	≥16	949	8 (NR)	79.3 (5.0)	63.6	MODERATE
Tyas et al. [56]	2001	Canada	N-ADRDA	MMSE	>26	CES-D20	≥16	694	3 to 5	65	67	MODERATE
Vilalta-Franch et al. [57]	2013	EU	DSM-IV	CAMCOG	≥79	DSM-IV	-	451	5	76.7 (5.4)	63.7	HIGH
Wilson et al. [58]	2003	USA	N-ADRDA	VARIOUS	-	CES-D10	≥4	142	3.9 (NR)	81.0 (6.6)	52.3	HIGH

Note: Meta-analyses analyzed were: Cherbuin et al. [24], Diniz et al. [22], Gao et al. [23], Kuring et al. [20], Kuring et al. [59], Santabárbara et al. [21]. ^1^ AD: Alzheimer’s disease. DSM-III-R, DSM-IV, DSM-V = Diagnostic and Statistical Manual of Mental Disorders; N-ADRDA = National Institute of Neurological and Communicative Disorders and Stroke–Alzheimer’s Disease and Related Disorders Association; N-AIREN = National Institute of Neurological and Communicative Disorders and Stroke–Association Internationale pour la Recherche et l’ Enseignement en Neurosciences; ICD-10 = International Classification of Diseases. Total of diagnoses are *k* = 30. ^2^ Cognitive measures: CERAD/K: Consortium to Establish a Registry for Alzheimer’s Disease; MMSE: Mini-Mental State Examination; CRS: Clinical Rating Scale; BIMC: Blessed Information-Memory-Concentration; CASI: Cognitive Abilities Screening Instrument; MSQ: Mental Status Questionnaire; CAMCOG: Cambridge Cognitive Examination. ^3^ Depression. DSM-III, DSM-III-R, DSM-IV, DSM-V: Diagnostic and Statistical Manual of Mental Disorders; HRSD17: Hamilton M. Rating Scale for DP; GMS-AGECAT: Geriatric Mental State-Automated Geriatric Examination for Computer Assisted Taxonomy; GDS-15/30: Geriatric Depression Scale; CES/-D10 (10 items)/- D11 (11 items)/-D20 (20 items) = Center for Epidemiologic Studies–DP Scale. ^4^ Cut-off criteria for categorial depression measures: HRSD-17, Hamilton et al. [60]; Williams et al. [61]; GMS-AGECAT, Copeland et al. [62]; GDS 15/30, Jung et al. [63]; Yesavage et al. [64]; SGDS, Kim et al. [65]; CES-D/D20, Radloff [66]; CES-D10/11, Kohout et al. [67]. ^5^ Follow-up: Total sample size for controls and healthy indicated; separate sample sizes for those with AD and depression and healthy controls were not reported. Study based on registry data. ^6^ AMSTAR 2 identifies quality of randomized controlled clinical trials. Rating overall confidence in the results: High = Zero or one non-critical weakness; Moderate = More than one non-critical weakness; Low = One critical flaw with or without non-critical weaknesses; Critically low = More than one critical flaw with or without non-critical weaknesses, Shea et al. [27] (https://amstar.ca/Amstar_Checklist.php accessed on 19 April 2021).

**Table 2:** Summary details for individual studies that examined the risk of dementia (OR) associated with depression.

Study Name	Statistics for Each Study					Exposed (AD)/Total	Exposed (AD)/Total
	*Odds Ratio*	*Lower Limit*	*Upper Limit*	*Z-Value*	*p-Value*	Cases (Depression)	Controls (No Depression)
Bae et al. [33]	9.84	0.57	170.00	1.57	0.116	9/359	0/181
Bartolini et al. [34]	16.00	3.72	68.76	3.73	<0.001	31/124	2/98
Becker at al. [18]	1.33	0.49	3.65	0.56	0.578	*HR* = 1.33 (0.49–3.65)	
Blasko et al. [35]	1.09	0.77	1.53	0.47	0.637	77/242	122/406
Burke et al. [36]	4.15	3.49	4.94	15.98	<0.001	205/1214	507/10,869
Chen et al. [37]	3.37	1.33	8.54	2.56	0.011	6/52	28/751
Dal Forno et al. [38]	2.63	1.28	5.40	2.63	0.008	*HR* = 2.63 (1.28–5.40)	
Devanand et al. [39]	5.07	3.02	8.52	6.13	<0.001	57/173	25/283
Dotson et al. [40]	1.02	0.77	1.35	0.11	0.911	96/938	125/1239
Fuhrer et al. [41]	1.04	0.69	1.58	0.19	0.849	30/203	196/1373
Gatz et al. [16]	3.49	1.08	11.28	2.09	0.037	*OR* = 3.49 (1.08–11.28)	
Geerlings et al. [42]	2.21	1.09	4.48	2.20	0.028	*OR* = 2.21 (1.09–4.48)	
Geerlings et al. [43]	1.41	0.55	3.58	0.71	0.475	6/35	44/343
Gracia-García et al. [44]	1.81	0.98	3.36	1.89	0.059	13/452	51/3174
Heser et al. [45]	2.70	1.80	4.03	4.84	<0.001	34/306	118/2663
Irie et al. [46]	9.94	3.16	31.22	3.93	<0.001	6/146	6/1397
Kim et al. [47]	1.33	0.62	2.85	0.74	0.463	*HR* = 1.33(0.62–2.85)	
Kim et al. [48]	2.33	0.97	5.56	1.90	0.057	7/45	38/518
Lauriola et al. [49]	130.73	7.90	2162.50	3.40	0.001	57/115	0/66
Lenoir et al. [50]	1.01	0.69	1.49	0.05	0.960	*HR* = 1.0 (0.7–1.6)	
Li et al. [51]	1.43	1.05	1.94	2.28	0.022	*HR* = 1.43 (1,05–1,94)	
Luchsinger et al. [52]	3.40	1.46	7.90	2.85	0.004	*HR* = 3.4 (1.5–8.1)	
Reding et al. [53]	19.00	12.42	29.06	13.59	<0.001	*HR* = 19.00 (12.40–27.90)	
Richard et al. [54]	2.03	1.44	2.86	4.06	<0.001	55/452	109/1708
Saczynski et al. [55]	1.76	1.03	3.01	2.07	0.039	*HR* = 1.76 (1.03–3.01)	
Tyas et al. [56]	2.00	1.01	3.95	2.00	0.046	21/36	271/658
Vilalta-Franch et al. [57]	2.36	1.11	5.03	2.23	0.026	13/116	17/335
Wilson et al. [58]	1.33	1.01	1.76	2.01	0.044	*OR* = 1.33 (1.01–1.76)	
**Random effects**	2.46	1.81	3.35	5.72	<0.001		

Note: AD: Alzheimer’s disease; NO-AD: No Alzheimer’s disease. Ns are based on total participant data available for depression or AD (not entire sample). Some data (N at baseline and follow-up) were not available for the depression and control groups, because studies did not provide them. In those cases, we reported the effect given in primary studies.

**Table 3:** Summary effect sizes.

		Model Statistics						
	*k*	*OR*	*LL*	*UL*	*Z*	*p*	*Q_W_*	*Q_B_*
Depression criteria (model 1)								
Clinic	11	3.68	2.44	5.55	6.20	0.0001	172.78 ***	6.86 **
Symptomatic	17	1.81	1.30	2.53	3.51	0.0001		
Depression scale (model 2)								
GDS	2	1.63	0.64	4.15	1.03	0.303	37.83 ***	1.87
CES-D	14	1.60	1.28	2.02	4.07	0.0001		
HSRD	1	3.40	1.19	9.71	2.29	0.022		
Cut-off (CES-D) (model 3)								
≥4	2	1.63	0.97	2.78	1.80	0.072	28.63 **	1.97
≥10	3	2.02	1.14	3.60	2.39	0.017		
≥16	8	1.44	1.04	2.00	2.19	0.028		
≥20	1	2.63	0.97	7.11	1.91	0.057		

Note: *** *p* ≤ 0.001, ** *p* ≤ 0.01, k: number of studies; OR: Odds ratio; LL: Lower limit; UL: Upper limit; Q_w_: heterogeneity within; Q_b_: heterogeneity between.
